# Depth and Well Type Related to Groundwater Microbiological Contamination

**DOI:** 10.3390/ijerph13101036

**Published:** 2016-10-21

**Authors:** Nayara Halimy Maran, Bruno do Amaral Crispim, Stephanie Ramirez Iahnn, Renata Pires de Araújo, Alexeia Barufatti Grisolia, Kelly Mari Pires de Oliveira

**Affiliations:** 1 Faculty of Exact Sciences and Technology, Federal University of Grande Dourados, Dourados, MS 79804-970, Brazil; nayaramaran@ufgd.edu.br (N.H.M.); brunocrispim.bio@gmail.com (B.d.A.C.); alexeiagrisolia@ufgd.edu.br (A.B.G.); 2 Faculty of Health Sciences, Federal University of Grande Dourados, Dourados, MS 79804-970, Brazil; stephanieiahnn@hotmail.com; 3 Faculty of Biological and Environmental Science, Federal University of Grande Dourados, Dourados, MS 79804-970, Brazil; renataaraujo@ufgd.edu.br

**Keywords:** total coliforms, *Escherichia coli*, wells, microbial resistance, water resources

## Abstract

Use of groundwater from private wells in households has increased considerably, owing to a better cost/benefit ratio than that of water provided by local utilities for a fee. However, this water is usually untreated, which makes it a vehicle for diseases. Thus, monitoring this water is necessary to ensure its integrity and quality. We aimed to evaluate the physical, chemical, and microbiological parameters of untreated groundwater drawn from different types of wells, and the antimicrobial susceptibility profile of the bacteria isolated from this water. Wellwater samples were collected in two Brazilian cities. Although physical and chemical parameters of the water were suitable for drinking, *Escherichia coli* was detected in 33% of the samples. *E. coli* contaminated 65% of dug wells and 10.25% of drilled wells. Many bacteria isolated were resistant to multiple antibacterial agents, including β-lactams. Microbial contamination of this water was related to the well depth, and was more common in dug wells, making this water unfit for human consumption. Consumption of such contaminated and untreated water is a public health concern. Thus, individuals who regularly use such water must be alerted so they may either take preventive measures or connect to the water distribution system operated by local utilities.

## 1. Introduction

Water can be a major route for spreading disease, especially among children, the elderly, and immunocompromised patients [1,2]. Diarrhea is the most common disease associated with the intake of water contaminated by pathogens through the fecal-oral route [3]. According to the World Health Organization [4], approximately 600,000 children die from diarrhea related to lack of water, sanitation, and hygiene. Contaminated water can cause bacterial infections, outbreaks [5], or serious damage to health, as well as social and economic losses [6]. Water contamination by multidrug-resistant microorganisms is also a concern, as this is a source of the spread of antimicrobial resistance [7].

According to the Brazilian National Water Agency [8], 86% of Brazilian cities are supplied by sources managed by municipal utilities tasked with supplying water to the city. Of this total, 44% use groundwater as their source, and 56% use surface water. Even if there is a municipal water supply network in these regions, residents sometimes use groundwater from individual wells for consumption. This is due to the low cost of well construction, ease of access to groundwater and, mainly, because there are no fees for groundwater use.

Consumption of groundwater and well drilling without suitable conditions increase the vulnerability to contamination, related either to anthropogenic or to natural activities. Degradation and contamination of groundwater are related to the depth and the type of well [9,10], to the presence of waste dumps and cemeteries, to improper well maintenance or abandonment, and to the use of septic tanks [11,12].

The lack of groundwater quality monitoring and of regulations governing the drilling of wells result in the population consuming water that lacks proper treatment. Consumption of water without adequate quality control is a public health concern, because it is often a vehicle for spreading disease. This study aimed to evaluate microbiological parameters of groundwater used for human consumption as they relate to well characteristics, and the antimicrobial susceptibility profile of the bacteria isolated.

## 2. Materials and Methods

### 2.1. Study Area and Well Types

Caarapó and Itaporã, located in the state of Mato Grosso do Sul, Brazil, where agropastoral activities are prevalent, have tropical climates and are situated above the Guarani Aquifer. Caarapó (Latitude: −22.6298; Longitude: −54.8253; 22°37’47”S, 54°49’31”W, elevation 470.2 m) has an estimated population in 2015 of 28,437 inhabitants, and a population density of 12.33 inhabitants/km^2^ [13]. The geology of the area includes the formation Serra Geral, a Mesozoic unit with basaltic rocks, and the Caiuá formation, comprising sandstone rocks, which are very porous and easily disintegrated [14]. Itaporã (Latitude: −22.0821; Longitude: −54.7889; 22°4’56”S, 54°47’20”W, elevation 350.3 m), with 22,896 inhabitants estimated in 2015, and a population density of 15.79 inhabitants/km^2^ [13], is in the Serra Geral geological formation.

The cities of Caarapó and Itaporã are supplied with treated water from artesian wells, and these systems are managed by municipal utilities. However, some households, often inhabited by older residents, choose to use water drawn from private wells, due to the residual chlorine taste in the treated water, with the belief that the quality of well water is superior, and, especially, to avoid paying fees.

The homes included in this study were selected with the help of local health workers and the criteria for choice were the wells whose groundwater was used for drinking. From July to October 2014, water samples from 66 domestic wells, 36 in Caarapó and 30 in Itaporã were collected and analyzed.

**Figure 1:**
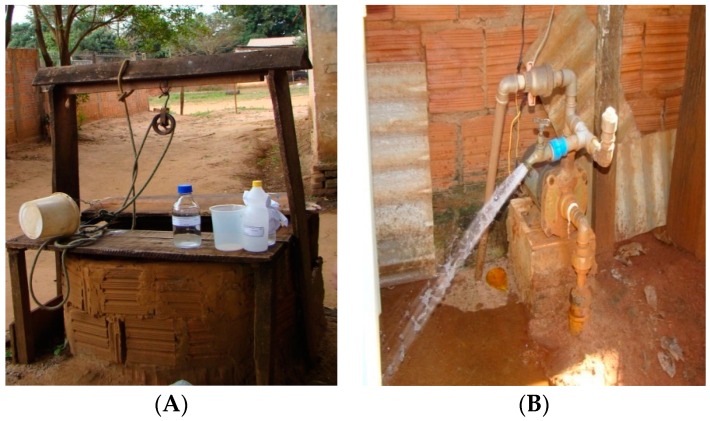
Types of wells analyzed. (**A**) Picture of a dug well; (**B**) Picture of a drilled well.

The wells were classified into either drilled wells or dug wells. A dug well is manually dug, with a diameter of about 1 m, and is popularly known as a *caipira*, *cacimba*, *rudimentar*, or *amazonas* (Figure 1A). A drilled well is made with machines or hand tools. The water is drawn by an external or submerged pump (Figure 1B).

### 2.2. Physico-Chemical Analyses

The physico-chemical analyses were carried out on location. Temperature and pH were measured with a multiparameter probe YSI Professional Plus (YSI Incorporated, Yellow Springs, OH, USA). Turbidity analysis was conducted with a portable turbidimeter (Policontrol), and chlorine and fluorine were measured by the colorimetric technique Alfakit [15].

### 2.3. Microbiological Analyses

Water samples were collected in sterile 500 mL glass bottles containing 0.5 mL of 1.8% sodium thiosulfate (Alfakit). For drilled wells, the water outlets were previously disinfected with 70% alcohol (*v*/*v*), and the water pump was turned on to draw water for 5 min before collection [15]. In dug wells, sample collection was carried out by submerging the glass bottle into the well [16]. Samples were transported under refrigeration (4 °C) and microbiological analysis was performed within 8 h of collection.

Total coliforms and *E. coli* were determined to be present or absent using Colilert [17] chromogenic and fluorogenic substrate, according to the manufacturer’s instructions. The spread-plate method was used for counting heterotrophic bacteria: samples were inoculated onto Plate Count Agar (HiMedia Laboratories, Mumbai, IND), either undiluted or at a 10^−1^ dilution, in duplicate, and were incubated at 35 ± 0.5 °C for 48 ± 2 h [18].

For yeast investigation, 100 mL of water sample was filtered through a cellulose ester membrane (Millipore 0.45 μm) in a pre-sterilized filtration assembly, then was placed on Sabouraud agar surface (HiMedia Laboratories, Mumbai, IND)with 50 mg/mL chloramphenicol, incubated at 37 ± 0.5 °C, and examined daily for seven days [19].

### 2.4. Identifying Heterotrophic Bacteria and Testing for Antimicrobial Susceptibility

Three colony-forming units (CFU) with different morphological characteristics from each PCA plate were selected and plated on blood agar (Newprov, Pinhais, PR, Brazil) and incubated at 35 ± 0.5 °C, to obtain pure cultures. After 24 ± 2 h they were Gram stained and a bacterial suspension McFarland 0.5, was prepared for identification with the VITEK 2 COMPACT (BioMérieux, Marcy l’Etoile, France) automated system, in duplicate at two different times.

Gram-positive organisms were identified with the AST-105 card (BioMérieux, Marcy l’Etoile, France), and resistance profile was determined with AST-P585 (BioMérieux, Marcy l’Etoile, France). For Gram-negative bacteria identification, AST-NG cards (BioMérieux, Marcy l’Etoile, France) were used, and AST-N239 (BioMérieux, Marcy l’Etoile, France) was used for determining antimicrobial resistance profile. The reliability threshold for identifying microorganisms was above 97% [20] and 16 standard antimicrobial agents were used for evaluating the resistance profiles.

For Gram-positive bacteria isolates, the antimicrobials used were: ampicillin (0.5, 4, 8, 32 μg/mL), benzylpenicillin (0.125, 0.25, 1, 2, 8, 64 μg/mL), ciprofloxacin (1, 2, 4 μg/mL), clindamycin (0.5, 1, 2 μg/mL), erythromycin (0.25, 0.5, 2 μg/mL), fusidic acid (0.5, 1, 4 μg/mL), gentamicin (8, 16, 64 μg/mL), linezolid (0.5, 1, 2 μg/mL), moxifloxacin (0.25, 2, 8 μg/mL), norfloxacin (0.5, 1, 4 μg/mL), oxacillin (0.5, 1, 2 μg/mL), rifampicin (0.25, 0.5, 2 μg/mL), teicoplanin (1, 4, 8, 16 μg/mL), tigecycline (0.25, 0.5, 1 μg/mL), trimethoprim/sulfamethoxazole (2/38, 8/152, 16/304 μg/mL), and vancomycin (1, 2, 4, 8, 16 μg/mL). For Gram-negative isolates: amikacin (8, 16, 64 μg/mL), ampicillin (8, 16, 64 μg/mL), ampicillin/sulbactam (4/2, 16/8, 32/16 μg/mL), cefepime (2, 8, 16, 32 μg/mL), cefoxitin (8, 16, 32 μg/mL), ceftazidime (1, 2, 8, 32 μg/mL), ceftriaxone (1, 2, 8, 32 μg/mL), cefuroxime (2, 8, 32 μg/mL), ciprofloxacin (0.5, 2, 4 μg/mL), colistin (4, 16, 32 μg/mL), ertapenem (0.5, 1, 6 μg/mL), gentamicin (4, 16, 32 μg/mL), imipenem (1, 2, 6, 12 μg/mL), meropenem (0.5, 2, 6, 12 μg/mL), piperacillin/tazobactam (2/4, 8/4, 24/4, 32/4, 32/8, 48/8 μg/mL), and tigecycline (0.75, 2, 4 μg/mL). Bacteria resistant to two or more antimicrobials were classified as multidrug resistant.

## 3. Results

### 3.1. Physico-Chemical Parameters

Of the 66 wells tested (see in Supplementary Materials Table S1), 60 had acidic water (pH below 6.5), and only two wells had turbidity values greater than 5.0 NTU (Nephelometric Turbidity Units). Free residual chlorine and fluorine were detected in six and 17 water samples, respectively, but within values suitable for consumption (Table 1).

### 3.2. Isolated Microorganisms and Antibiotic Resistance

Total coliforms contaminated 67% of the samples and *E. coli*, 33%. Itaporã had *E. coli* contamination in 50% of the wells, and Caarapó in 19.4% (Table 2).

The amount of microorganisms varied according to well depth. In wells up to 5 m deep, 90% showed contamination by total coliforms, 60% by *E. coli*, 70% by yeast, and 20% of wells displaying a heterotrophic bacteria count greater than 500 CFU/mL (Table 3).

Six Gram-positive and 39 Gram negative were isolated from all the wells analyzed and those with antimicrobial resistance are listed in Table 4, with the greatest resistance to β-lactams displayed by bacteria.

## 4. Discussion

Physico-chemical parameters are essential for monitoring the treatment of drinking water in distribution systems [21,23,24], but no control standards exist for private wells. The low pH observed in these samples may be due to the Latossol soil of these regions, which is acidic [14,25], and which may cause corrosion of the water-conducting pipes [21].

The high temperature of some samples favors microorganism growth and alters their organoleptic characteristics, such as taste, odor, and color [21,22]. Elevated levels of turbidity may be caused by suspended particles, or by the very nature of the rocks [26], or due to improper pipe maintenance, which is a problem for consumers using well water.

The residual chlorine detected in wells results from the addition of sodium hypochlorite at 2.5% (25,000 ppm) by health workers in an attempt to perform water treatment, but without any specific criteria. As a result, the improper use of chlorine fails to protect residents from groundwater contamination, as the appropriate course would be to perform the treatment after extracting the water, which is not what residents are being instructed to do.

The fluorine concentration in the samples was low, as reported in other studies [27,28] and the fluorine present may originate from the type of rock (Latossol) in the region [29]. The importance of determining fluorine content must be stressed, as the intake of high concentrations of this substance can stain the teeth and, in severe cases, cause crippling skeletal fluorosis, whereas, if present in water at appropriate amounts, it helps to prevent dental caries [23].

Microbiological contamination of water can result in disease transmission [21]. The presence of *E. coli* in 33% of the wells was similar to contamination rates reported in other studies [30,31]. However, 67% of dug-type wells had *E. coli* contamination. Factors such as diameter of the well opening [9], contact buckets, submerged motor pumps, and anthropogenic activities may have favored the contamination of dug wells. In addition to these factors, these wells (which are up to 5 m deep) are more susceptible to contamination due to their proximity to the soil surface.

The city of Itaporã had a higher number of dug wells because the water table is shallower there than in Caarapó, which had the highest number of drilled wells, indicating a deeper water table. Thus, we found that a shallower well depth and the use of dug wells correlates with greater microbiological contamination of groundwater (Table 2 and Table 3).

The detection of heterotrophic bacteria may indicate contamination [21,32]. Yeast has the ability to adhere and form biofilms in the pipes and hoses of pressure pumps used in households for groundwater extraction, producing substances that can cause unpleasant tastes and odors [24]. Even though the WHO 2011 recommendation does not require analysis of heterotrophic bacteria and yeast, our survey showed that these microbes displayed behavior similar to total coliforms and *E. coli*, where shallower wells increase the possibility of microbial contamination.

The proportion of antibiotic-resistant bacteria and multi-drug resistance (>2 antibiotics) phenotypes in this search were 26.7% and 64.4%, respectively. Among the antibiotics tested, 91.1% of resistance belongs to β-lactams. This result indicated that antimicrobial-resistant bacteria in water suggest the presence of pathogenic organisms that may spread infection among groundwater consumers [16], especially affecting children, the elderly, and immunocompromised people. Comparatively, other studies in surface water and wastewater treatment plants also detected the presence of microorganisms resistant to antibiotics [33,34]. Studies suggest that the selection of resistant microorganisms occurs because of human waste that is released into the natural environment, causing a serious ecological problem [33].

*Klebsiella* spp., *Enterobacter* spp., *Citrobacter* spp., *Serratia* spp., *Chromobacterium violaceum*, *Pseudomonas aeruginosa*, and *Acinetobacter* spp., which were isolated in this analysis, have also been observed in similar studies [16,31]. These microbes, identified in well water, are indeed relevant, as they are related to nosocomial agents.

The antimicrobial resistance profiles of the isolates were similar to those of microorganisms isolated from clinical samples [35,36,37] and the environment-hospital-environment dissemination of these microorganisms should be considered. The presence of *E. coli* in water indicated the existence of contamination from sewage, which could increase the chance that antibiotic-resistant pathogens could be transmitted via this route [38]. The presence of bacteria resistant to first- and second-line drugs, such as β-lactams, in drinking water can aggravate the condition of immunocompromised patients by limiting therapy options, as infections develop more rapidly in these patients. Human activity can cause the spread of resistant microorganisms, potentially resulting in the dissemination of multidrug-resistant agents in hospitals, the community, and the environment, and thus is considered a public health problem [7].

## 5. Conclusions

The physico-chemical measurements did not preclude water consumption according to the WHO. Regarding microbiological conditions, contamination is directly related to the use of dug and shallow wells.

Microbiological data in this study indicate that, besides *E. coli*, disturbing antimicrobial-resistant bacteria were also present. This finding highlights the need for sanitation improvements in order to prevent microorganism contamination, including immediate intervention through public health policies related to preventing contamination, and deployment of control plans for regulating the quality of water from private wells, including disabling wells whose conditions are improper.

Furthermore, the population consuming water from wells should take preventive measures, such as boiling the water, using water purifiers, or connecting to the local utility’s water distribution system, in order to avoid health risks.

## Figures and Tables

**Table 1:** Physico-chemical parameters of groundwater from private wells in the cities of Caarapó and Itaporã, MS, Brazil (2014).

Physico-Chemical Parameters		Caarapó				Itaporã				Total *n* = 66		Reference Values
		Dug *n* = 10		Drilled *n* = 26		Dug *n* = 17		Drilled *n* = 13				
		*n*	%	*n*	%	*n*	%	*n*	%	*n*	%	
**pH**	<6.5	7	70	25	96	16	94	12	92	60	91	6.5–8.5 *
	6.5–8.5	3	30	1	4	1	6	1	8	6	9	
**Temperature**	15.1–24 °C	7	70	6	23	5	29	1	8	19	29	24.2 °C **
	>24 °C	3	30	20	77	12	71	12	92	47	71	
**Turbidity**	≤5.0 NTU	10	100	26	100	16	94	12	92	64	97	≤5.0 NTU *
	>5.0 NTU	0	0	0	0	1	6	1	8	2	3	
**Chlorine**	<0.1 mg/L	8	80	24	92	16	94	12	92	60	91	≤5.0 mg/L *
	0.1–1.25 mg/L	2	20	2	8	1	6	1	8	6	9	
**Fluorine**	<0.1 mg/L	7	70	17	65	16	94	9	69	49	74	≤1.5 mg/L *
	0.1–0.65 mg/L	3	30	9	35	1	6	4	31	17	26	

* WHO, 2011 [21]; ** Values above 24.2 °C suggest potential for microorganism growth [22].

**Table 2:** Number of wells positive for total coliforms and *E. coli* in the cities of Caarapó and Itaporã, MS, Brazil (2014).

Microorganisms	Caarapó						Itaporã						Totals *	
	Dug *n* = 10		Drilled *n* = 26		Total *n* = 36		Dug *n* = 17		Drilled *n* = 13		Total *n* = 30			
	*n*	%	*n*	%	*n*	%	*n*	%	*n*	%	*n*	%	*n*	%
**Total Coliforms**	10	100	7	27	17	47.2	16	94	7	54	23	76.6	40	67
***E. coli***	6	60	1	4	7	19.4	12	71	3	23	15	50	22	33

* Contamination present in 100 mL sample [21].

**Table 3:** Relationship between well depth and contamination with total coliforms, *E. coli*, heterotrophic bacteria, and yeast.

Depth	Total Number of Wells	Total Col. *		*E. coli* *		Heterotrophic **		Yeast *	
		*n*	%	*n*	%	*n*	%	*n*	%
≤5 m	10	9	90	6	60	2	20	7	70
>5–10 m	19	15	79	9	48	2	11	10	53
>10–15 m	16	10	63	6	38	1	6	8	50
>15 m	21	6	28	1	5		-	5	24
Total	66	40	60	22	33	5	8	30	45

*n* = wells number; * Presence in 100 mL of water; ** Counts above 500 colony-forming units. Interpretation according to Resolution 2914/2011 Brazil.

**Table 4:** Antimicrobial resistance and identification by the VITEK system of 45 bacteria isolated from well water.

**Microorganism**	**N**	**β-Lactams**		**LI**		**MA**	**QU & FL**			**AM**	**OX**		**AN**	**SU & PR**	**GP**		
**Gram positive**		**P**	**OXI**	**CM**		**E**	**CIP**	**NOR**	**MXF**	**GM**	**LNZ**		**RA**	**SXT**	**TEC**		**VA**
*Staphylococcus epidermidis*	1	S	S	S		S	S	S	S	S	S		S	R	S		S
*Staphylococcus hominis* ssp.	2	R	S	S		R	S	S	S	S	S		S	R	S		S
*Staphylococcus saprophyticus*	1	R	R	S		S	S	S	S	S	S		S	R	S		S
*Staphylococcus warneri*	2	R	S	S		R	S	S	S	S	S		I	R	I		S
**Gram negative**	**N**	**β-lactams**											**FU**	**QU & FL**	**AM**		**GC**
		**ETP**	**AM**	**SAM**	**FEP**	**FOX**	**CAZ**	**CRO**	**CXM**	**IPM**	**MEM**	**TZP**	**CS**	**CIP**	**AN**	**GM**	**TGC**
*Acinetobacter haemolyticus*	4	-	R	R	S	R	I	R	R	S	S	R	S	R	S	S	-
*Acinetobacter lwoffii*	1	-	R	S	S	R	I	R	R	S	S	S	S	S	S	S	-
*Aeromonas hydrophila*	2	-	-	-	S	-	S	S	-	S	S	S	S	S	S	S	-
*Chromobacterium violaceum*	1	-	-	-	S	-	S	R	-	I	-	R	R	S	S	S	-
*Citrobacter freundii*	2	S	R	S	S	R	S	S	R	S	S	S	-	S	S	S	-
*Citrobacter sedlakii*	1	S	R	S	S	R	S	S	R	S	S	S	-	S	S	S	-
*Enterobacter asburiae*	2	S	R	S	S	R	S	S	R	S	S	S	-	S	S	S	-
*Enterobacter cloacae complex*	2	S	R	R	S	R	S	S	R	S	S	S	-	S	S	I	-
*Enterobacter gergoviae*	1	S	R	S	S	S	S	S	R	S	S	S	-	S	S	S	-
*Klebsiella oxytoca*	1	S	R	S	S	S	S	S	S	S	S	S	-	S	S	S	-
*Klebsiella pneumoniae* spp.	1	S	R	S	S	S	S	S	S	S	S	S	-	S	S	S	-
*Kluyvera intermédia*	1	S	S	S	S	S	S	S	S	S	S	R	-	R	S	S	-
*Ochrobactrum anthropi*	1	-	-	-	S	-	R	R	-	S	S	R	S	R	S	I	-
*Pantoea* spp.	1	-	-	-	S	-	S	S	-	S	S	-	-	S	S	S	-
*Pseudomonas aeroginosas*	5	-	R	R	S	R	S	R	R	S	I	S	S	S	S	S	R
*Pseudomonas putida*	1	-	-	-	S	-	S	I	-	S	S	I	S	S	S	S	-
*Ralstonia mannitolilytica*	1	-	-	-	S	-	S	S	-	R	I	R	R	S	S	R	-
*Serratia marcescens*	10	S	R	R	S	R	S	S	R	I	S	S	R	S	S	S	-
*Sphingomonas paucimobilis*	1	-	-	-	I	-	R	I	-	S	S	S	R	R	S	S	-

R—Resistant, I—Intermediate, S—Susceptible—Interpretation according to the CLSI (Clinical and Laboratory Manual Standards Institute) 2014; N—number of bacteria isolated. Group of Antibiotic = LI: Lincosamides, MA: Macrolides, QU & FL: Quinolones & Fluoroquinolones, OX: Oxazolidinones, AM: aminoglycosides, AN: ansamycins, GP: Glycopeptides, SU & PR: sulfonamides & Primidinas, GC: glycylcyclines, FU: Fusidanas, PO: Polymyxins; Antibiotic = P: Penicillin, AM: Ampicillin, OXI: oxacillin, SAM: Ampicillin/Sulbactam, FEP: Cefepime, FOX: Cefoxitin, CAZ: Ceftazidime, CRO: Ceftriaxone, CXM: Cefuroxime, ETP: Ertapenem, IPM: Imipenem, MEM: Meropenem, TZP: Piperacillin/Tazobactam, CM: Clindamycin, E: Erythromycin, CIP: Ciprofloxacin, MXF: Moxifloxacin, NOR: Norfloxacin, LNZ: Linezulide, GM: Gentamicin, AN: Amikacin, RA: Rifampicin, TEC: Teicoplanin, VA: Vancomycin, SXT: trimethoprim/sulfamethoxazole, TGC: Tigecycline, FA: Fusidic acid, CS: Colistin.

## Supplementary Materials

The following are available online at www.mdpi.com/1660-4601/13/10/1036/s1, Table S1: Description of the characteristics of the 66 wells tested.
