# Supplementary material for: Depth and Well Type Related to Groundwater Microbiological Contamination
Source: Int J Environ Res Public Health. 2016 Oct 21;13(10):1036. doi: 10.3390/ijerph13101036 (PMC5086775; doi:10.3390/ijerph13101036)
Supplement: Supplementary file 1 [file ijerph-13-01036-s001.pdf]

Supplementary Materials: Depth and Well Type Related to Groundwater Microbiological Contamination

Nayara Halimy Maran, Bruno do Amaral Crispim, Stephanie Ramirez Iahnn, Renata Pires de Araújo, Alexeia Barufatti Grisolia and Kelly Mari Pires de Oliveira

Table S1. Description of the characteristics of the 66 wells tested.

Cities	Wells	Chlorine (mg/L)	Fluorine (mg/L)	Turbidity (NTU)	pH	Temperature (°C)	Depth (m)	Type of Wells	Total Coliform	*E. coli*	Heterotrophic Bacteria (CFU)
Itaporã	1	0	0	<0.10	5.82	25.9	9	Drilled	Present	Absent	1
	2	0	0.14	<0.10	7.24	24.4	30	Drilled	Absent	Absent	4
	3	0	0	<0.10	5.56	24.1	8	Drilled	Present	Absent	5
	4	0	0	<0.10	6.2	24.5	6	Dug	Present	Absent	5
	5	0	0	<0.10	6	23.8	6	Dug	Present	Present	260
	6	0	0	<0.10	4.78	23.8	3	Dug	Present	Absent	70
	7	0	0	<0.10	4.98	24.4	3	Dug	Present	Present	>500
	8	0	0	5.3	4.97	25.5	10	Dug	Present	Present	400
	9	0	0	<0.10	6.5	22.5	6	Dug	Present	Present	11
	10	0.74	0	<0.10	5.8	25.3	12	Dug	Absent	Absent	0
	11	0	0.64	<0.10	5.31	25.2	13.5	Drilled	Absent	Absent	0
	12	0	0	<0.10	5.34	25.5	4	Dug	Present	Present	401
	13	0	0	<0.10	5.8	24.4	13	Dug	Present	Present	49
	14	0	0	<0.10	5.5	23.5	8	Dug	Present	Present	189
	15	0	0	<0.10	6.04	24.7	15	Drilled	Present	Present	1
	16	0	0	<0.10	6.15	24.5	8	Drilled	Present	Absent	1
	17	0	0	<0.10	6.45	21.9	12	Drilled	Present	Present	1
	18	0	0	<0.10	5.62	24.1	6	Drilled	Absent	Absent	1
	19	0	0.1	<0.10	5	24.4	4.2	Dug	Present	Present	112
	20	0	0.12	<0.10	4.87	24.9	7	Drilled	Absent	Absent	3
	21	0	0.21	<0.10	5.12	24.5	5	Drilled	Absent	Absent	2
	22	0	0	<0.10	6.16	25.6	75	Drilled	Absent	Absent	0
	23	0	0	<0.10	6.14	23.3	9	Dug	Present	Present	>500
	24	0.36	0	15.8	5.48	25.5	70	Drilled	Absent	Absent	1
	25	0	0	<0.10	5.67	25.7	4.5	Dug	Present	Present	404

	26	0	0	<0.10	5.3	24.7	13	Dug	Present	Absent	23
	27	0	0	<0.10	5	24.7	10	Drilled.	Present	Present	17
	28	0	0	<0.10	5.15	24.7	14.5	Dug	Present	Absent	89
	29	0	0	<0.10	5.4	25.5	12	Dug	Present	Present	2
	30	0	0	<0.10	5.96	24.1	4	Dug	Present	Present	101
Caarapó	1	0.1	0	<0.10	7.32	24.3	4.5	Dug	Present	Absent	33
	2	0	0	<0.10	4.86	22.6	16	Drilled	Absent	Absent	9
	3	0	0	<0.10	5.46	25.9	14	Drilled	Present	Absent	7
	4	0	0	<0.10	5.67	25.3	4	Dug	Present	Absent	35
	5	0	0	<0.10	7	24.1	12	Dug	Present	Present	39
	6	0.18	0	<0.10	6.24	24.6	14	Drilled	Absent	Absent	1
	7	0	0.29	<0.10	5.48	24.2	29	Drilled	Absent	Absent	0
	8	0	0	<0.10	4.83	24.6	7	Drilled	Absent	Absent	1
	9	0	0.25	<0.10	5.8	23.9	13	Drilled	Absent	Absent	1
	10	0	0.25	<0.10	5.7	25.5	12	Drilled	Absent	Absent	2
	11	0	0.55	<0.10	4.95	24	20	Drilled	Absent	Absent	0
	12	0	0.32	<0.10	5.35	23.6	8	Dug	Present	Present	14
	13	0	0	<0.10	6.7	24.4	20	Drilled	Absent	Absent	1
	14	0	0.32	<0.10	5.31	25.4	19.5	Drilled	Absent	Absent	0
	15	0	0.45	<0.10	5.84	24.8	10	Drilled	Absent	Absent	0
	16	0	0	<0.10	5.08	24.8	20	Drilled	Absent	Absent	0
	17	0	0.09	<0.10	6.57	21.5	7	Dug	Present	Present	56
	18	0	0	<0.10	5.55	24.8	30	Drilled	Absent	Absent	0
	19	0	0.31	<0.10	6.23	24.4	13	Drilled	Absent	Absent	0
	20	0	0	<0.10	6.25	23.9	19	Drilled	Present	Present	136
	21	0	0.03	<0.10	5.39	24.6	21	Drilled	Absent	Absent	4
	22	0	0.03	<0.10	5.5	23.9	21	Drilled	Present	Absent	232
	23	0	0	<0.10	5.63	24.8	22	Drilled	Absent	Absent	3
	24	0	0.09	<0.10	5.5	23.5	8	Dug	Present	Absent	55
	25	0	0	<0.10	5.42	22.7	20	Drilled	Present	Absent	16
	26	0	0	<0.10	5.3	21.9	15	Dug	Present	Present	>500
	27	0.23	0	<0.10	4.86	24.5	19	Drilled	Absent	Absent	22
	28	1.23	0	<0.10	5	22.5	5	Dug	Present	Present	187
	29	0	0	1.06	4.95	23.6	7	Dug	Present	Absent	300

30	0	0	<0.10	4.74	25.3	30	Drilled	Present	Absent	2
31	0	0	<0.10	5.08	25.1	18	Drilled	Absent	Absent	0
32	0	0	<0.10	5.63	23.4	8	Dug	Present	Present	>500
33	0	0	<0.10	5.03	24.6	20	Drilled	Absent	Absent	1
34	0	0	<0.10	4.93	24.6	18	Drilled	Absent	Absent	2
35	0	0	<0.10	5.15	24.4	14	Drilled	Present	Absent	3
36	0	0	<0.10	4.75	24.4	27	Drilled	Present	Absent	7

© 2016 by the authors; licensee MDPI, Basel, Switzerland. This article is an open access article distributed under the terms and conditions of the Creative Commons by Attribution (CC-BY) license (http://creativecommons.org/licenses/by/4.0/).
